# Bilateral proptosis as the first manifestation in a patient with acute myeloid leukemia: Case report

**DOI:** 10.1097/MD.0000000000047767

**Published:** 2026-02-28

**Authors:** Ruba Edrees, Mary Hazeem, Maged Kheder

**Affiliations:** a Department of Pediatrics, Children’s University Hospital, Faculty of Medicine, Damascus University, Damascus, Syrian Arab Republic; b Department of Hematology and Oncology, Children’s University Hospital, Faculty of Medicine, Damascus University, Damascus, Syrian Arab Republic; c Pediatrics Hematopoietic Stem Cell Transplantation Center, National Stem Cell Center (HAYAT), Children’s University Hospital, Faculty of Medicine, Damascus University, Damascus, Syrian Arab Republic.

**Keywords:** acute myeloid leukemia, childhood cancer, chloroma, granulocytic sarcoma, myeloid sarcoma, orbit, proptosis

## Abstract

**Rationale:**

Myeloid sarcoma (MS), also known as granulocytic sarcoma or chloroma, is a rare manifestation of acute myeloid leukemia (AML) that occurs concurrently with, following, or rarely before the onset of leukemia. It consists of immature myeloid cells that proliferate in extramedullary tissues. Owing to the rare association between bilateral proptosis and AML, it is often misdiagnosed as other conditions that present with proptosis, such as infections (as in our case), vascular lesions, or tumors, particularly rhabdomyosarcoma and neuroblastoma, creating a diagnostic dilemma.

**Patient concerns:**

We report a case of MS that initially presented with bilateral proptosis and was misdiagnosed as orbital cellulitis. A child of Syrian origin presented with rapidly progressive bilateral proptosis that had started 2 weeks prior to admission. Her medical history was unremarkable except for a blood transfusion 2 months earlier. She initially received treatment for suspected conjunctivitis and then for orbital cellulitis with intravenous antibiotics, with no clinical improvement. On examination, she appeared pale and fatigued. Ophthalmic examination revealed severe bilateral eyelid edema with proptosis and conjunctival chemosis.

**Diagnoses and interventions:**

MS was diagnosed based on laboratory tests, bone marrow examination, and computed tomography imaging. A complete response was achieved dramatically after 1 month of treatment with the standard chemotherapy regimen.

**Outcomes:**

This case highlights the various aspects of AML in children and aims to draw clinicians’ attention to the possible diagnosis of AML in a patient with proptosis, which may lead to earlier diagnosis and treatment.

**Lessons:**

This case raises awareness among clinicians to consider AML in the differential diagnosis of any child with unilateral or bilateral proptosis, even in the absence of systemic symptoms of leukemia.

## 1. Introduction

Acute leukemia is the most common childhood cancer, accounting for approximately 31% of all malignancies occurring in children younger than 15 years.^[1]^

Acute myeloid leukemia (AML) accounts for 15% to 20% of childhood leukemia cases, whereas acute lymphoblastic leukemia accounts for the majority of cases (80%).^[2]^

Myeloid sarcoma (MS), also known as granulocytic sarcoma or extramedullary myeloid tumor (previously called chloroma), is a rare manifestation of AML that occurs in only 2.5% to 9.1% of patients, either concurrently with, following, or rarely before the onset of leukemia.^[3,4]^

Although it can occur at any site throughout the body, the skin and orbit are the most common sites in children.^[5–7]^

We report a case of an infant with bilateral orbital exophthalmos that eventually led to AML with MS diagnosis; this patient was treated with a standard chemotherapy regimen and responded dramatically within 1 month.

## 2. Case report

A 1.5-year-old girl was brought to our outpatient department hospital complaining of pallor during the past 2 months, with a positive history of blood transfusion at a remote center to correct acute anemia (hemoglobin level (Hb) = 5 g/L) without further evaluation due to limited diagnostic resources.

Later, the patient developed a rapidly progressing bilateral protrusion in both eyes 2 weeks prior, which was initially treated as conjunctivitis and then as orbital cellulitis with intravenous antibiotics without improvement.

There was no history of fever, malaise, poor appetite, weight loss, recurrent infections, or easy bleeding.

On arrival, the patient appears pale and fatigued with no external signs of bruising.

She was alert, and her physical signs were normal for her age. Findings from her heart and lung examinations were normal. Her abdominal examination had normal findings, without hepatomegaly or splenomegaly. Her neurologic examination had normal findings.

**Figure 1.**
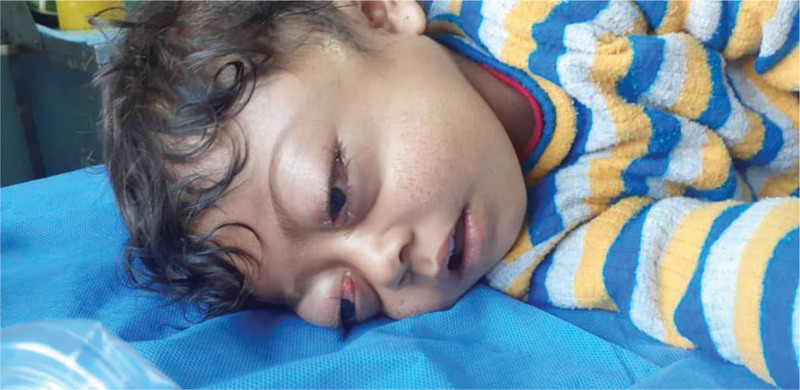
At admission, severe bilateral eyelid edema with proptosis, incomplete eyelid closure, and conjunctival chemosis are evident.

Eye examination revealed severe bilateral eyelid edema with proptosis, incomplete eyelid closure, and conjunctival chemosis (Fig. 1). There was no restriction in eyeball movement or relative afferent papillary defects.

Fundus examination revealed bilateral first-degree optic nerve papilledema with retinal vascular congestion.

Upon arrival, laboratory studies were performed, and a complete blood count revealed a white blood cell count (WBC) of 30,000 × 10^9^/L with a predominance of neutrophils. Her Hb level was 11.8 g/dL, and her platelet count was 158,000 × 10^9^/L.

Uric acid, renal, and liver function tests were all normal.

The erythrocyte sedimentation rate was 82 mL/hour in the first hour, and the lactate dehydrogenase concentration was 2125 U/L.

Owing to the high WBC, erythrocyte sedimentation rate, lactate dehydrogenase levels, and history of a new blood transfusion, a fast peripheral blood smear examination was performed, revealing an elevated WBC count with a predominance of blast cells that urgently alter bone marrow aspiration.

**Figure 2.**
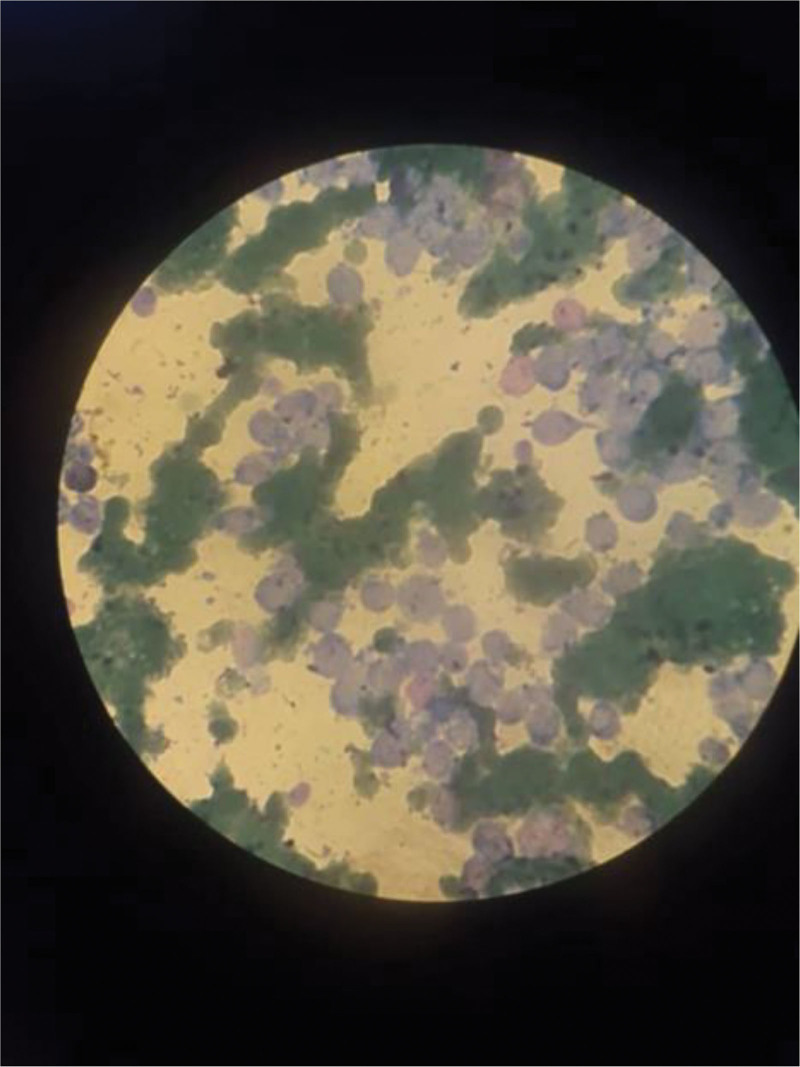
Bone marrow aspiration (BMA) examination revealed hypercellular bone marrow infiltrated with large cell blasts, which could be myeloid.

Bone marrow aspiration examination revealed hypercellular bone marrow (BM) infiltrated with large cell blasts, which could be myeloid (Fig. 2). Then, flow cytometry analysis of the BM samples was performed.

Later, the patient developed clinical deterioration the next day, with dyspnea, fever, and poor oral intake. Laboratory tests revealed hyperleukocytosis, with WBC counts of 112,000 × 10^9^/L, and she was urgently managed with leukapheresis in combination with prophylaxis for tumor lysis syndrome (intravenous fluids and allopurinol).

Clinical stability and cytoreduction were achieved within 3 days in conjunction with the results of flow cytometry analysis, which revealed a hypercellular BM with 84% myeloblasts, showing positivity for CD45, CD4, CD64, CD117, and CD38, which is consistent with AML M5.

**Figure 3.**
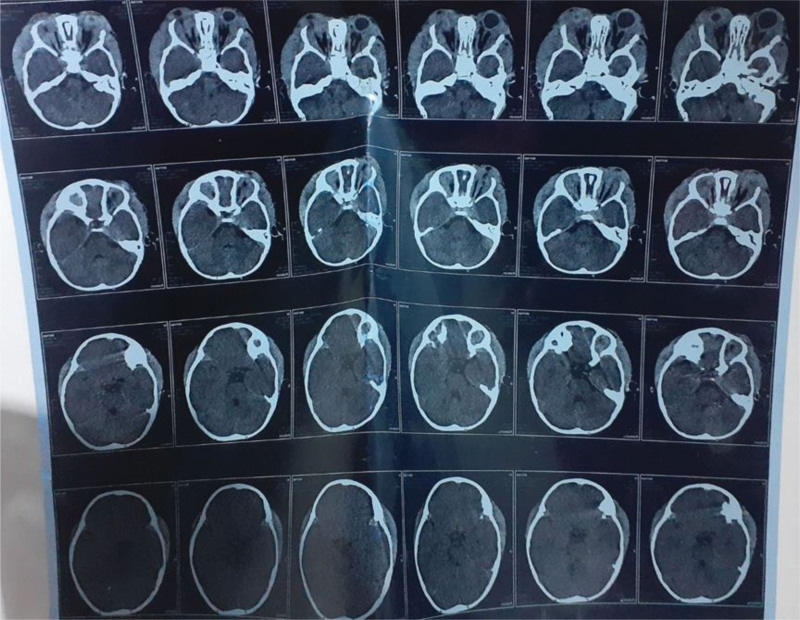
CT scan of the head and orbit revealed anterior displacement of the right eye globe without bone erosion. CT = computed tomography.

A computed tomography scan of the head and orbit revealed a bilateral posterolateral orbital mass with focal necrosis causing anterior displacement of the right eye globe without bone erosion (Fig. 3).

Based on the clinical, laboratory, and imaging results, a diagnosis of AML with orbital MS was made.

The induction therapy phase was initiated 5 days after admission and consisted of intravenous cytarabine (200 mg/m^2^/day) for 5 days in combination with mitoxantrone (12 mg/m^2^/day) for 3 days, followed by the first phase of consolidation therapy (after 21 days of induction), which consisted of 1000 mg/m^2^/day cytarabine and 20 mg/m^2^ adriamycine for 3 days.

Lumbar puncture was performed on the first day of induction, and cerebrospinal fluid analysis was normal.

The first prophylactic dose of intrathecal cytarabine was given.

BM reexamination was performed at the end of the first part of the consolidation phase (after a month of treatment), which revealed complete remission.

**Figure 4.**
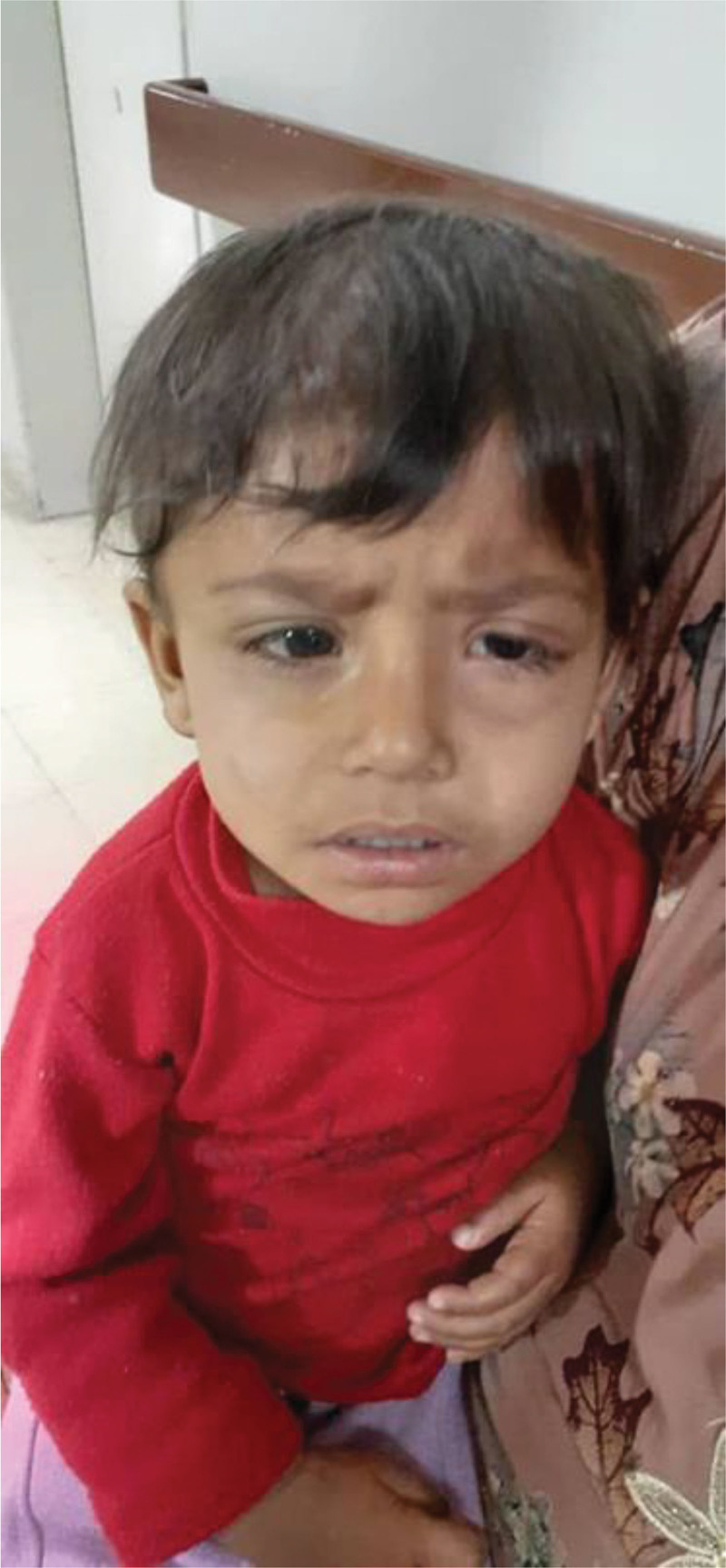
At the end of the first part of the consolidation phase: significant retrogression of orbital involvement.

In fact, remission was ensured clinically by complete retrogression of orbital involvement (Fig. 4).

However, the patient was lost to follow-up thereafter.

## 3. Discussion

MS is recognized as the presence of proliferating myeloid blasts at any site outside the BM that disrupts the architecture of normally involved tissues.^[8]^

They are thought to accumulate in the BM and then migrate through the haversian canals to the subperiosteum, leading to the formation of soft tissue masses.^[9]^

This explains why they are commonly observed in bones, especially in the skull (and more especially the orbit), where they manifest as proptosis due to active hematopoiesis at these sites.^[10,11]^

Other manifestations include ptosis, eyelid edema, decreased vision, diplopia, and extraocular movement restriction.^[12]^

Shields et al published a review of the literature among many reported cases of orbital MS and reported that approximately 60% of orbital MS cases are bilateral and that 88% of them manifest as proptosis with no history of leukemia at the time of diagnosis.^[13]^ Our patient complained of bilateral eyelid edema with proptosis and severe chemosis without vision problems. She did not have symptoms suggestive of leukemia except for a previous history of blood transfusion to correct severe anemia (Hb = 5 g/dL).

However, our patient had already developed hyperleukocytosis within 3 days of admission.

Orbital MS is known to be associated with the French-American-British AML subtypes M2, M4, and M5,^[14]^ which is consistent with our case.

The most common differential diagnoses that should be excluded include orbital infections and tumors, including Burkitt lymphoma, Ewing sarcoma/primitive neuroectodermal tumors, rhabdomyosarcoma, and neuroblastoma metastases.^[15,16]^

The diagnosis of MS with AML is easier than that of isolated MS, which creates a diagnostic dilemma.^[17,18]^

However, owing to its rarity as a variation of AML, MS can be misdiagnosed with other diseases.

Meis et al reported in a retrospective series that the misdiagnosis rate of MS without evidence of acute leukemia was 75%.^[17]^

Razem et al described a case of orbital MS in a child with blunt eye trauma, which was misdiagnosed as orbital subperiostal hematoma.^[18]^ In the same context, our patient was first treated for conjunctivitis and then for orbital cellulitis.

The diagnostic tools must include different imaging techniques and histopathological and immunohistochemical analyses of blood, BM, and, if possible, tumor tissue.^[19,20]^

In our case, the diagnosis of AML with MS was made via BM aspiration analysis and computed tomography imaging, and once the diagnosis was made, treatment was initiated.

Available therapeutic options include chemotherapy, radiotherapy, surgery, and hematopoietic stem cell transplantation.^[7]^

Given the nature of AML as a systemic disease, chemotherapy is still the optimal treatment in most cases. Cytarabine-containing induction and consolidation chemotherapies have been the most common treatment options.^[7]^

Our patient was started on induction therapy, with cytarabine and mitoxantrone, followed by consolidation therapy with high-dose cytarabine and adriamycin.

One month after chemotherapy, a remarkable improvement was observed clinically, and the BM examination revealed complete remission.

## 4. Conclusion

This case illustrates an atypical manifestation of AML in children, which raises awareness among clinicians to keep it in mind in any child with unilateral or bilateral proptosis, even in the absence of systemic symptoms of leukemia.

A blood smear and subsequent BM examination should be considered for early diagnosis and treatment.

## Author contributions

**Data curation:** Ruba Edrees.

**Supervision:** Maged Kheder.

**Writing – original draft:** Ruba Edrees.

**Writing – review & editing:** Ruba Edrees, Mary Hazeem.
